# Development and Assessment of a New Empirical Model for Predicting Full Creep Curves

**DOI:** 10.3390/ma8074582

**Published:** 2015-07-22

**Authors:** Veronica Gray, Mark Whittaker

**Affiliations:** Institute of Structural Materials, Swansea University, Singleton Park, Swansea SA1 8PP, UK; E-Mail: m.t.whittaker@swansea.ac.uk

**Keywords:** creep, modelling, lifing

## Abstract

This paper details the development and assessment of a new empirical creep model that belongs to the limited ranks of models reproducing full creep curves. The important features of the model are that it is fully standardised and is universally applicable. By standardising, the user no longer chooses functions but rather fits one set of constants only. Testing it on 7 contrasting materials, reproducing 181 creep curves we demonstrate its universality. New model and Theta Projection curves are compared to one another using an assessment tool developed within this paper.

## 1. Introduction

Over the last 50 years, creep modelling has become an area of significant expertise as components are being designed with more precision for increasingly extreme environments. As such, engineers and scientists have endeavored to understand, describe and predict the phenomenon of creep focusing on the rupture time and minimum strain rate of a material [1]. Simply predicting these two material properties is no longer sufficient as components are being designed to operate under specific constraints beyond these two points on the creep curve [2]. As is, few models exist that do this effectively, with the most well-known being Theta Projection [3], Wilshire Equations [4,5] and Uniaxial Creep Lifing [6].

These creep curve models at some point in their implementation require a user to define functions from data trends [1]. This is what gives these models flexibility but also makes them strongly subjective with each user’s interpretation of the data defining the model [7]. This paper describes a model where this subjective or “experienced eye” is no longer needed. By removing the need for a user to choose functions it is hoped a standardised approach to modelling creep curves is possible.

In developing a model the goal is it for it to be universally applicable. This means the model should be able to reproduce creep curves for an array of materials over a range of conditions. It thus raises the question of “how many materials and conditions must it reproduce accurately before the model can be accepted?” To address this, the new model has been applied to seven materials including a pure element and a range of alloys with different compositions, crystal structures and microstructures. The new model was used to reproduce 181 creep curves sourced from Swansea University’s historical database covering tests conducted from the early 1990s through to the current day.

Developing a new model requires the ability to assess how accurate it is. There is no standardised approach to judging how accurate a creep model is compared to real data. As such, there are a range of curve shapes and vast differences in magnitudes, meaning a combined quantitative-qualitative approach needs to be used. One has been developed here with pass/fail criteria and applied to the new model. A case study using Theta Projection as a comparison is also provided.

## 2. Assessing Curve Fit

**Figure 1:**
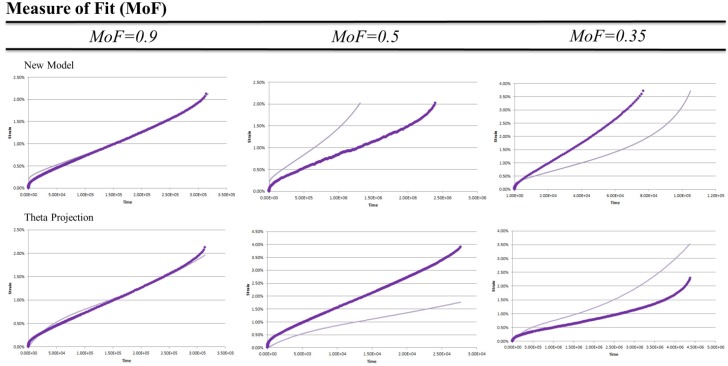
Examples of Measure of Fit Values for New Model and Theta Projection on linear scale. Experimental data (♦) model prediction (▬).

Numerically defining the fit of a function is a complex problem as there are many ways of doing so requiring a balance between quantitative and qualitative errors [8,9,10]. The Z parameter currently recommended by the ECCC, is given as Z = 10^2.5S^ where S is the standard deviation of the residual log time [11]. This measure gives a quantitative approach on a logarithm scale which does not necessarily reflect the nature and magnitude of the whole curve, and does not provide as clear a comparison to select acceptability criteria. Fits produced by any creep model need to be judged both on qualitative and quantitative error and as such a consistent numerical “Measure of Fit” (MoF) was developed. The MoF combines two error measurements with the Pearson correlation coefficient or R^2^ value reflecting the qualitative fit [8,9,10], and the percentage difference reflecting the quantitative fit.

(1) $$MoF=R^{2}\left(1-\%Diff\right)$$

(2) $$R^{2}=\frac{\sum^{\text{​}}(x-\bar{x})\left(y-\bar{y}\right)}{\sqrt{\sum^{\text{​}}{\left(x-\bar{x}\right)}^{2}\sum^{\text{​}}{\left(y-\bar{y}\right)}^{2}}}$$

(3) $$\%Diff=\frac{\sum^{\text{​}}\left(y-x\right)}{\sum^{\text{​}}y}$$

where *y* is the measured data and *x* the predicted value and correspondingly $\bar{y}$ and $\bar{x}$ are the mean of these values. By this method a MoF = 1 indicates a perfect fit. Figure 1 shows a range of curve fits from both the New Model and Theta Projection on a linear scale.

**Figure 2:**
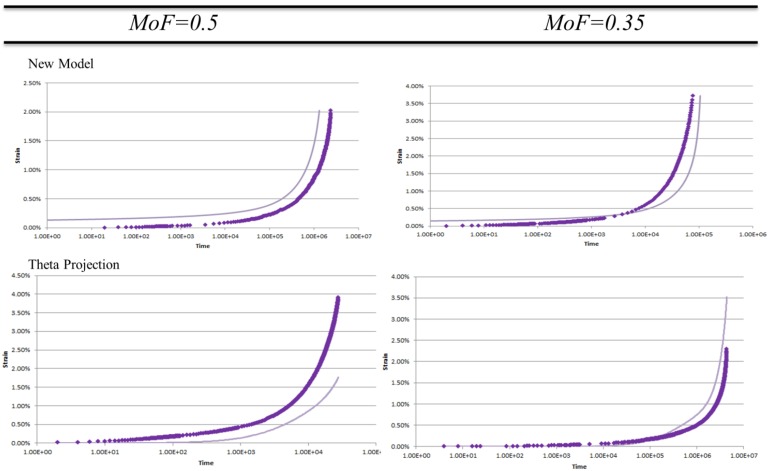
Examples of MoF = 0.5 & 0.35 for New Model and Theta Projection on logarithmic scale. Experimental data (♦) model prediction (▬).

Looking at Figure 1 it can be seen for a MoF = 0.9 the curve closely resembles the data. There are minor differences due mainly to shape rather than magnitude. The mismatch of shape at different points in the curve for different methods shows the qualitative nature of the measure in that shape errors at higher values do not necessarily produce lower MoF. The MoF = 0.5 show curves which either have magnitude errors as seen in the New Model, or, have shape error as seen in Theta Projection where the tertiary upturn is missing. A MoF = 0.35 shows both the shape and magnitude contribute to the low value describing a model that neither reflects the values or trends of the actual data. As creep data is often displayed on a logarithmic scale, the MoF = 0.5 & 0.35 are shown in Figure 2 on a logarithmic scale.

It is from the more classical representation of data seen in Figure 2, that the pass/fail criteria of MoF = 0.5 was chosen. Looking at MoF = 0.5 we can see the error stems from consistent mistmatch in either magnitude as for the New Model example, or, shape in the Theta Projection example (lacking tertiary upturn, see Figure 1). These errors show the model reflects at least some significant aspects within the data, and as such are reproducing some features of the real creep curves. For an MoF = 0.35 both magnitude and shape of the predictions do not match the actual data thus there is little correlation between the model and reality. Given this, the pass/fail criteria was chosen to be an MoF > 0.5.

## 3. Description of New Model

This model utilises the normalised activation energy, Q_c_^*^ [12]. Unlike the traditional activation energy of *Q_c_* obtained at constant σ, the normalised activation energy is obtained at constant σ/σ_N_, where σ_N_ can be the temperature dependant Ultimate Tensile Strength (UTS), Proof Stress, or Yield Strength. This approach to activation energy was used as it provides a more complex link between stress and temperature, and has been found to provide values more consistent with proposed creep mechanisms than the traditional *Q_c_* [4,5,12,13]. In the analysis undertaken here, UTS was used to normalise stress.

The underlying premise of this model is the observation from numerous creep datasets that for a given strain, the time to strain is related to the normalised stress by a power relationship:

(4) t(ε)exp(−Qc*RT)=M(ε)(1−σσN)P(ε)

For a given strain, the time to strain *t(ε) vs.* normalised stress *(1 −* σ/σ_N_*)* collapses into a master curve for all stress-temperature combinations. This master curve can be described by a power equation of the form *y = M(ε)·x^P(ε)^* (M = multiplier, P = power) as seen in Equation (4). Examination of the values for *M* and *P* over progressive strains showed that these values can be reproduced for each strain using a Frechet and Lognormal distribution with μ = 0, such that:

(5) $$M\left(\varepsilon \right)=A_{1}exp\left(-{\left(\frac{\varepsilon }{A_{2}}\right)}^{-A_{3}}\right)$$

(6) $$P\left(\varepsilon \right)=\;\frac{A_{4}}{\varepsilon A_{5}\sqrt{2\pi }}exp\left(-\frac{{\left[ln\left(A_{6}\varepsilon \right)\right]}^{2}}{2A_{5}^{2}}\right)+\;A_{7}$$

where *A_1_-A_7_* are constants obtained from fitting the defined functions *M(ε)* and *P(ε)*. If the constants are known, then for a given stress-temperature combination the whole strain curve can be predicted.

## 4. Results

Taking the raw creep dataset for a material, the normalised activation energy is obtained from the gradient of *ln(t_f_) vs. 1/T* for σ/σ_N_ = constant. For 0.1, 0.2, 0.3, 0.5, 0.7, 1, 2, 3, 4 and 5% strains, the time to strain was extracted from the creep curves. For each strain, t(ε)exp(−Q_c_^*^/RT) vs. (1 − σ/σ_N_*)* was graphed and a power based trend line of the form *y = Mx^P^* was fitted. The values for *M(ε)* and *P(ε)* at the specific strains were then fitted to the previously mentioned functions and all constants obtained.

### 4.1. Inconel 100

**Figure 3:**
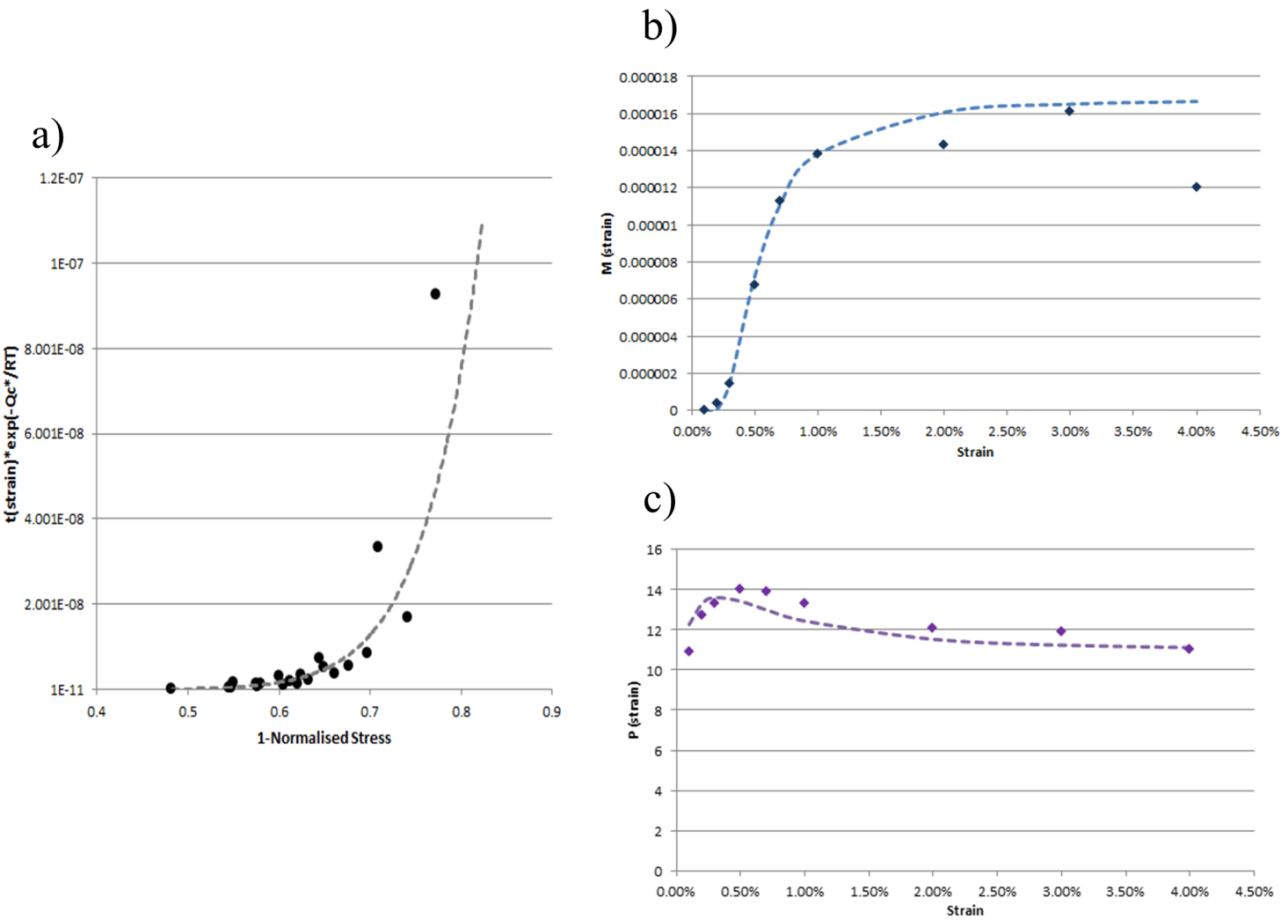
Determination of model constants: (**a**) t(ε)exp(−Q_c_^*^/RT) vs. (1 − σ/σ_N_*)* fitted with function of form *y = Mx^P^*(dotted line) for a given strain; (**b**) coefficient *M(ε) vs. ε* fitted with function in Equation (5) (dotted line); (**c**) coefficient *P(ε) vs. ε* fitted with function in Equation (6) (dotted line).

Inconel 100 is used in this article to provide a detailed case study of the new model. It is a material with a composition of 15% wt Co, 10% wt Cr, 5.5% wt Al, 4.5% wt Ti, 3% wt Mo, 1% wt V, 0.18% wt C, 0.06% wt Zr, 0.014% wt B, balance Ni. A total of 22 creep tests ranging over stresses of 150–400 MPa and temperatures of 1073–1223 K were available from Swansea University’s historical database. Taking the raw data from tests, the time to 0.1, 0.2, 0.3, 0.5, 0.7, 1, 2, 3, 4% strain was extracted. For each strain, t(ε)exp(−Q_c_^*^/RT) vs. (1 − σ/σ_N_*)* was graphed and a power based trendline of the form *y = Mx^P^* was fitted. The values for *M(ε)* and *P(ε)* at these strains were then fitted to Equations (5) and (6). Figure 3 shows these steps graphically. From these two fits all seven constants were determined for IN100 (A_1_ = 1.68 × 10^−5^, A_2_ = 0.0046, A_3_ = 2.09, and A_4_ = 4, A_5_ = 1, A_6_ = 110, A_7_ = 11).

Having obtained all the constants, the model can now be used to reproduce creep curves with the user input being normalised stress and temperature values. Each predicted curve was then evaluated by the MoF. As a further means of comparison the Theta Projection method was used on the same data and the MoF evaluated. The form of the Theta Projection function was taken from Harrison *et al.* [14] with Q_2_^*^ = Q_4_^*^ = Q_c_^*^, A_1_ = 0.0114, A_2_ = 4.66 × 10^10^, A_3_ = 0.0981, A_4_ = 4.69 × 10^9^, n_1_ = 0.92, n_2_ = 5.29, n_3_ = 3.29, and n_4_ = 4.91. The results of both creep prediction methods are shown in Table 1.

**Table 1:** IN100 MoF for new model and theta projection. Failures highlighted in grey text (MOF < 0.5).

Stress (MPa)	Temperature (K)	Normalised Stress	MoF: New Model	MoF: Theta Projection
350	1073	0.3501	0.941	0.705
375		0.3751	0.927	0.827
400		0.4001	0.709	0.379
425		0.4251	0.770	0.553
450		0.4501	0.497	0.016
300	1123	0.3384	0.774	0.863
325		0.3666	0.896	0.522
350		0.3948	0.607	0.776
375		0.4230	0.854	0.861
400		0.4512	0.907	0.900
200	1173	0.2587	0.679	0.253
225		0.2910	0.914	0.867
250		0.3234	0.764	0.847
275		0.3557	0.462	−10.528
300		0.3881	0.890	0.603
325		0.4204	0.809	0.448
350		0.4527	0.352	0.537
400		0.5174	0.772	0.487
150	1223	0.2274	0.740	0.520
200		0.3032	0.744	0.654
250		0.3789	0.169	0.416
300		0.4547	0.804	0.778

Table 1 shows the results of both the New Model and Theta Projection in predicting whole creep curves for IN100. It can be seen that the New Model has four failures and Theta Projection eight failures. Interestingly, three of the New Model failures coincide with failures by Theta Projection. Given no account has been taken for data scatter, it is possible these results are likely to stem from the reality of testing rather than from model failure as neither model predicts them. Removing such curves from the dataset was considered, but choosing which data follows trends is subjective and therefore all curves have been included.

**Figure 4:**
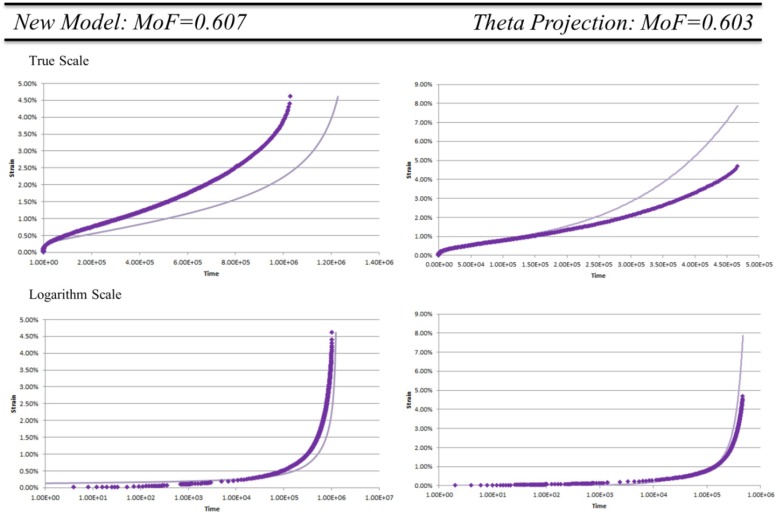
Comparison of New Model and Theta Projection for MoF ≈ 0.6. Experimental data (♦) model prediction (▬).

To further illustrate the differences between methods we consider the lowest common pass MoF of the methods. For the New Model this is MoF = 0.607, and so for Theta Projection MoF = 0.603. These are shown in Figure 4 on a true and logarithm scale.

From Figure 4 we can see that both these models reproduce the general shape of the creep curves with the transitions from primary to steady state to tertiary creep in the same locations as the data. The error of the methods is traceable to either over or under prediction of tertiary creep. Looking at the MoF of data within the same temperature regimes the New Model appears to better represent the progression of the curves as all other MoF are significantly higher. For the Theta Projection the model has an unpredictable pass rate meaning the progression of the creep curves over stress is not well reflected.

### 4.2. Pure Copper

Pure copper is known to be a difficult material to creep life due to changes in mechanism and, in some cases, the appearance of sigmoidal primary creep. Applying the New Model to pure copper, the same process was followed where Q_c_^*^ and constants A_1_–A_7_ were determined (A_1_ = 8 × 10^4^, A_2_ = 0.004, A_3_ = 1.5, and A_4_ =10, A_5_ = 3, A_6_ = 5, A_7_ = 10.17). The MoF values for pure copper are shown in Table 2 with only one fit considered a fail.

**Table 2:** Pure copper MoF results. Failures highlighted in grey text (MOF < 0.5).

Stress (MPa)	Temperature (K)	Normalised Stress	MoF: New Model
55.2	608	0.4042	0.586
68.9		0.5045	0.901
75.8		0.5550	0.939
41.4	645	0.3416	0.625
55.2		0.4555	0.242
82.7		0.8624	0.857
34.5	688	0.3339	0.571
48.3		0.4675	0.718
52.8		0.5111	0.938
68.9		0.6669	0.728
27.6	728	0.3184	0.964
48.3		0.5572	0.506
27.6	774	0.4086	0.872
17.2	823	0.3646	0.906
20.7		0.4388	0.641
27.6		0.5850	0.517

**Figure 5:**
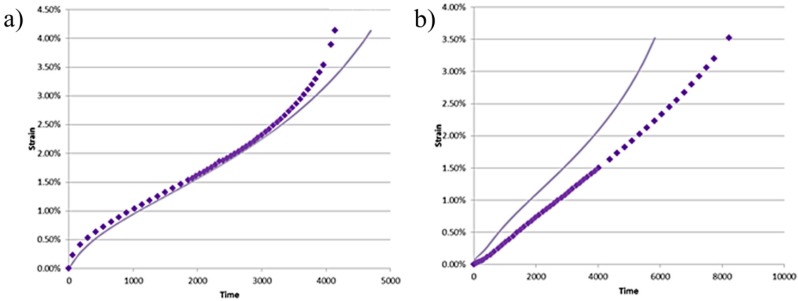
Pure Copper New Model Prediction: (**a**) 52.8 MPa 688 K; and (**b**) 27.6 MPa 774 K. Experimental data (♦) model prediction (▬).

The focus of this section is not on the accuracy of the New Model, but rather on a particular observation. Figure 5 shows the data and New Model prediction for 52.8 MPa 688 K and 27.6 MPa 774 K.

Looking at Figure 5a we see the classically shaped creep curve, in Figure 5b we see creep data that has sigmoidal or inverse primary creep. The interesting observation here is that the New Model reproduced this non-classical primary creep shape without modification. In practical terms the only difference between the two predicted curves is the input of normalised stress and temperature. This particular occurrence is highlighted as the understanding of inverse and sigmoidal primary creep is limited and as such the phenomena is very unpredictable [15]. If this model, through further work, is able to reliably predict non-logarithmic primary creep then this may change how we view and understand the process of primary creep itself.

### 4.3. Summary of Results

The performance of the New Model has been evaluated thoroughly and been compared to Theta Projection evaluated by MoF. The model reproducing non-logarithmic primary creep without modification for the case of pure copper has also been discussed. Having a good understanding of the model and its implementation we consider the seven materials and 181 creep curves as a full set of data.

Table 3 lists the success rates of the model in reproducing creep curves according to the MoF value. The materials examined are:

- Al2124. This material has a composition of 3.76% wt Cu, 1.33% wt Mg, 0.49% wt Mn, 0.08% wt Fe, <0.01% wt Zn, 0.02% wt Si, <0.02% wt Cr, and Al for the remaining balance.
- Al2419. This material has a composition of 6.06% wt Cu, 0.24% wt Mn, 0.13% wt Fe, 0.06% wt Si, 0.16% wt Zr, 0.01% wt Zn, 0.09% wt V, 0.09% wt Ti, 0.01% Mg, and Al for the remaining balance.
- Al7010. This material has a composition of 6.14% wt Zn, 2.43% wt Mg, 1.62% wt Cu, 0.13% wt Zr, 0.07% wt Fe, 0.01% wt Mn, 0.05% wt Si, 0.04% wt Ti, 0.01% Cr, and Al for the remaining balance.
- Al8090. This material has a composition of 2.34% wt Li, 1.14% wt Cu, 0.64% wt Mg, 0.1% wt Zr, 0.03 wt Fe, 0.02% wt Si, 0.02% wt Ti, <0.02% wt Zn, <0.01% Mn, <0.01% wt Ni, <0.01% wt Cr, and Al for the remaining balance.
- Pure Copper.
- IN100. The composition of this super alloy has already been stated above.
- NIM105. Nimonic 105 is a material with a composition of 20% wt Co, 14.85% wt Cr, 4.98% wt Mo, 4.79% wt Al, 1.23% wt Ti, <0.15% wt Si, 0.04% wt Mn, 0.01% wt Cu, 0.095% wt Zr, 0.125% wt C, 0.004% wt S, 0.006% wt B, <0.001% wt Pb, <0.001% wt Ag, <0.001% wt Bi, and the remaining balance Ni.

**Table 3:** Pass/Fail assessment of seven materials and 181 creep tests.

Material	Stress Range (MPa)	Temperature Range (K)	Pass	Fail	Total
Al2124	200–440	373–463	26	9	35
Al2419	150–300	373–463	13	10	23
Al7010	110–390	373–463	13	10	23
Al8090	130–410	373–463	14	6	20
Pure Copper	18–83	608–823	15	1	16
IN100	150–400	1073–1223	18	4	22
NIM105	58–400	1098–1223	26	16	42
–	–	*Total:*	*125*	*56*	*181*

The results seen in Table 3 show a good pass rate especially for Al2124, Al8090, Pure Copper, IN100 and NIM105. The poorer pass rate of Al2419 and Al7010 on closer inspection was expected as the initial collapsing of the data into a master curve produced more scatter than the other materials. If the MoF pass rate was dropped to 0.4 then the number of failed curves halves indicating that the model reproduces some aspects of the data but not with sufficient accuracy for the pass/fail criteria defined here. It is also important to note no account of data scatter has been taken into consideration as there is no widely accepted standard on creep curve scatter.

## 5. Discussion

Traditionally, creep curves are displayed on a logarithmic scale where primary creep is not easily recognisable. Using this representation often skews the visual perception of the accuracy of a model as logarithmic scale skews the data and suppresses much of the finer detail. With the desire to design to more precise conditions it is important to reproduce the creep curve and its features on the true scale.

In developing a model, the issues of “burden of proof” and how to measure accuracy have been addressed. Although some readers may disagree with the way it has been done here, it is important to note there is no established standard and very little advice onto how to proceed under these circumstances. Using IN100 the New Model was described and compared to the more familiar Theta Projection. Furthermore the incidental reproduction of sigmoidal primary creep of Pure Copper raised the idea that the model may be able to deal with non-traditional primary creep unlike other existing models.

In looking at this New Model, we can observe:

- This model hinges upon the power relationship between a time to strain and the normalised stress. How effective the model will be in predicting curves can be initially assessed from the data spread and variation from this initial power relationship.
- The second most obvious concern is the number of constants. Although there are a total of seven constants these are obtained simultaneously from only two fits *i.e.*, A_1_–A_3_ is obtained from a single line fit, and A_4_–A_7_ from another. Unlike many other creep models these constants once defined remain constant unless region splitting is observed.
- Although *P(ε)* appears to be a complex function, it is a common function which in MS Excel simplifies to “A_4_*LOGNORM.DIST(A_6_ε, 0, A_5_, FALSE) + A_7_”. On rare occasion multiple fits can be obtained for this function but incorrect values produced unphysical curves which were easily identified.
- The simplicity of the model is that the user only defines the normalised stress and temperature conditions to produce full strain-time creep curves.
- This model is based on a continuous function that does not terminate at fracture (t_f_), nor is easily differentiated to produce έ_min_. This model seeks to reproduce the whole curve rather than predicting two points within the curve.

It is acknowledged that this model is not a perfect physical description of creep, but is important as it adds another tool for design engineers to predict creep and advance our understanding of this phenomenon.

Future research and application of this model may include:

- Examining the range of temperatures and normalised stresses over which the power relationship holds. This will define the range over which this method can be used to predict whole creep curves. If this master curve is widely applicable then shorter tests may be sufficient to predict long term creep curves.
- The ability of this model to predict the transition in copper from logarithmic primary creep to sigmoidal needs further investigation. This needs to be examined across other materials and conditions to establish if this is reliable or specific to the case examined.
